# Constructing Hetero-Microstructures in Additively Manufactured High-Performance High-Entropy Alloys

**DOI:** 10.3390/e27090917

**Published:** 2025-08-29

**Authors:** Yuanshu Zhao, Zhibin Wu, Yongkun Mu, Yuefei Jia, Yandong Jia, Gang Wang

**Affiliations:** 1 State Key Laboratory of Materials for Advanced Nuclear Energy, Shanghai University, Shanghai 200444, China; zhaoyuanshu@shu.edu.cn (Y.Z.); wzb0907@shu.edu.cn (Z.W.); yuefeijia94@shu.edu.cn (Y.J.); g.wang@shu.edu.cn (G.W.); 2 Zhejiang Institute of Advanced Materials, Shanghai University, Jiashan 314100, China; 3 Institute of Materials, Shanghai University, Shanghai 200444, China

**Keywords:** high entropy alloys, additive manufacturing, gradient structure, layered structure, multi-level heterogeneous structure

## Abstract

High-entropy alloys (HEAs) have shown great promise for applications in extreme service environments due to their exceptional mechanical properties and thermal stability. However, traditional alloy design often struggles to balance multiple properties such as strength and ductility. Constructing heterogeneous microstructures has emerged as an effective strategy to overcome this challenge. With the rapid advancement of additive manufacturing (AM) technologies, their unique ability to fabricate complex, spatially controlled, and non-equilibrium microstructures offers unprecedented opportunities for tailoring heterostructures in HEAs with high precision. This review highlights recent progress in utilizing AM to engineer heterogeneous microstructures in high-performance HEAs. It systematically examines the multiscale heterogeneities induced by the thermal cycling effects inherent to AM techniques such as selective laser melting (SLM) and electron beam melting (EBM). The review further discusses the critical role of these heterostructures in enhancing the synergy between strength and ductility, as well as improving work-hardening behavior. AM enables the design-driven fabrication of tailored microstructures, signaling a shift from traditional “performance-driven” alloy design paradigms toward a new model centered on “microstructural control”. In summary, additive manufacturing provides an ideal platform for constructing heterogeneous HEAs and holds significant promise for advancing high-performance alloy systems. Its integration into alloy design represents both a valuable theoretical framework and a practical pathway for developing next-generation structural materials with multiple performance attributes.

## 1. Introduction

Since the middle of the 20th century, metallic materials have consistently held a dominant position in the materials industry. However, the strength and toughness of metallic materials are often mutually exclusive. Researchers have attempted to achieve a breakthrough by optimizing the composition, selecting the process, and implementing various treatment procedures to alter the internal microstructure of the materials in the hope of achieving both high strength and high ductility simultaneously. In recent years, individuals have deviated from the conventional paradigm of alloy design, combining five or more major elements in an equal atomic ratio and obtaining high-entropy alloy materials with ordered atomic arrangement but disordered element arrangement [1,2]. This alloy possesses characteristics such as thermodynamic high-entropy effect, kinetic slow diffusion effect, structural lattice distortion effect, and performance “cocktail” effect. Compared with traditional alloys, it has many significant advantages, including high strength and hardness, excellent corrosion resistance, outstanding thermal stability, and radiation resistance [1,2,3]. For some developed high-entropy alloys, These materials can concurrently achieve high strength and high plasticity, thus addressing the long-standing challenge associated with the mutually exclusive nature of these properties in conventional metals [4,5,6,7,8,9,10]. However, many key scientific issues regarding its deformation mechanism and the intrinsic correlation between structure and performance have not yet been clearly understood. Many studies are still at the stage of experimental verification. In response to the current industrial applications and the more demanding and complex working conditions, only by developing high-efficiency preparation solutions and solutions with scalable microstructures, especially regarding the formation of multi-level non-uniform structures of crystals and their influence and mechanism on the rational properties of alloys, can the new high-entropy alloy materials achieve significant development under the impetus of various engineering application backgrounds.

## 2. The Regulation of Performance by Minute Defects Within Crystals

The study of crystal structure regards each particle of the crystal, including molecules, atoms, and ions, as being arranged in a periodic and regular manner, which is an idealized model. However, most materials naturally have certain defects. The existence of intrinsic defects in crystals will inevitably affect the microscopic structure within the crystal, thereby further influencing the performance of the crystal material. Generally speaking, based on the size differences in the defects, all structural irregularities in solid materials are divided into four major categories: zero-dimensional point defects (such as vacancies and doping), one-dimensional line defects (such as helices and edge dislocations), two-dimensional planar defects (such as grain boundaries and twin boundaries), and three-dimensional volume defects (such as lattice disorder, vacancies, and second-phase precipitation). According to the atomic structure of the material, defects of different dimensions can be further subdivided into more types of defects. Theoretical studies have shown that the presence of defects can improve the mechanical properties, corrosion resistance, wear resistance, and high-temperature performance of metal materials. The introduction of multiple types of microstructural defects and how to control the microstructural defects will have a crucial impact on the performance of the material. In high-entropy alloys, the spatial composition of multiple elements is conducive to the development of new catalysts and the adjustment of surface adsorption properties, thereby reducing the demand for precious metals. The random mixture of multiple elements provides diverse active sites, which is particularly suitable for series catalysis. Its high heterogeneity provides multiple adsorption and reaction sites for molecular adsorption [11,12,13]. Meanwhile, the stability of the high-entropy structure improves the stability of the structure in harsh catalytic environments; for example, Qiu et al. prepared a nano-porous high-entropy alloy AlNiCuPtPdAu, and the effective oxidation-reduction reaction (ORR) of this nano-porous high-entropy alloy only decayed by 7.5% after 100,000 electrochemical cycles. At the same time, adjusting the composition of the high-entropy alloy can also regulate the hydrogen storage performance while keeping the basic structure unchanged, as Kao et al. demonstrated that the hydrogen storage performance can be adjusted by changing the content of Ti, V, and Zr in the CoFeMnTiVZr alloy. In the research of Leandro Serrano et al., there are a small amount of C14 and Laves phases between the dendrites within the TiVNbCrMn high-entropy alloy, and the appearance of these micro-precipitated phases enhances its hydrogen storage performance, indicating that the presence of a small amount of precipitated phases can improve the maximum hydrogen storage capacity of the alloy [14,15].

For the face-centered cubic structure high-entropy alloys, they usually exhibit high plasticity and relatively low strength. In order to further obtain strong and tough face-centered cubic structure high-entropy alloys to meet the application requirements in various fields, a controllable and efficient microstructure regulation method is needed to obtain the ideal microstructure configuration and promote the ideal microstructure replacement adjustment strategy of high-entropy alloys. However, for high-entropy alloys with the advantage of multi-component design, the diversity and complexity of their microstructure undergo significant changes depending on the types and ratios of the components [16]. The emergence of the characteristics of this structure is partly determined by the component factors, and the other part is also influenced by the preparation process [17,18,19]. Therefore, in addition to the design and optimization of the composition of high-entropy alloys, a more effective approach is to manipulate and reconfigure the microstructural defects of the original high-entropy alloys through process selection. This aims to find effective methods for regulating and reconfiguring the microstructure of high-entropy alloys, thereby establishing a theoretical correlation between microstructure and performance. This is an important goal pursued in the current field of high-entropy alloys for further expansion and accelerating its engineering application.

### 2.1. Research on Data-Driven Microstructure Prediction of High Entropy Alloys

In 2004, Yeh and Cantor, among others, proposed the concept of high-entropy alloys [20,21]. This new alloy design strategy focuses on the underexplored central regions of the multi-element phase diagram, where all the alloying elements are concentrated together without any significant base elements [21,22]. High entropy alloys are also regarded as a major breakthrough in alloying theory in recent years. Their preparation methods are diverse, and they can be produced in large quantities through traditional methods such as casting, forging, powder metallurgy, and additive manufacturing [18,23,24,25,26,27,28,29,30,31,32]. Due to their advantages in many aspects, including high strength, resistance to fatigue and fracture, thermal stability, wear resistance, corrosion resistance, radiation resistance, and other new physical properties, high-entropy alloys show great potential in the engineering field, such as metallurgical materials, catalysts, aerospace materials, and nuclear materials [3,33,34,35,36,37,38,39,40,41,42,43]. However, as a new type of multi-component metallic alloy material, high-entropy alloys have facilitated extensive exploration of unprecedented structural properties. Like many other metallic materials, the most promising applications of high-entropy alloys are believed to be in the field of structures. Therefore, a large amount of research has focused on their mechanical properties, mainly strength and ductility [44,45,46,47,48,49,50,51,52,53]. From an engineering perspective, high-entropy alloys have stimulated the exploration of the extensive configurational space provided by adjusting multiple major elements. The complex alloying and thermal mechanical processing can affect the microstructure of these alloys, thereby providing various excellent and industrially achievable mechanical properties.

The complexity of the composition combination of high-entropy alloys determines the great difficulty in exploring the components of high-performance high-entropy alloys. The differences in microstructures formed by different compositions, and how the interactions between each element affect the final structure, are all issues that must be considered in the research process. Obtaining high-entropy alloy components with high performance through experiments often relies on great randomness because, starting from the definition of high-entropy alloys, the almost endless combinations of components that may have high performance are possible. Traditional methods have significant limitations in finding materials with good functionality and mechanical properties. However, with the development of computer technology in the research of high-entropy alloy microstructures using data-driven methods, by combining advanced computational models with experimental verification, new perspectives have been provided for understanding the formation and stability of phases in complex alloy systems. Rao et al.’s research [54] proposed an active learning framework based on machine learning (ML) technology, applicable to high-entropy alloy composition prediction in sparse datasets. The model proposed in this study demonstrated efficient learning ability and prediction accuracy in predicting the thermal expansion coefficient of high-entropy alloys. The proposal of this model provides an effective tool for the discovery of high-performance high-entropy alloys. Shi et al. analyzed the formation mechanism of chemical short-range order (CSRO) in TiFeCoNi-based materials by combining first-principles methods, Monte Carlo (MC) methods, and machine learning-assisted methods, revealing the significant influence of the $t_{2g}-e_{g}\;$ orbital relationship between elements on CSRO [55]. This research further promoted the application of computer technology in the study of the complex structure of high-entropy alloys. Shuai Chen et al. explored the influence of SRO on the ultimate strength and ductility of CoCuFeNiPd alloys by combining Monte Carlo [56], molecular dynamics simulation, and density functional theory calculations. The study found that three clusters developed from SRO were face-centered cubic preferred (FCCP) clusters, insensitive clusters, and body-centered cubic preferred (BCCP) clusters. The insensitive cluster served as the matrix, the FCCP as the hard filler to enhance strength, and the BCCP cluster as the soft filler to increase ductility. This research emphasized the importance of SRO in affecting the mechanical properties of HEAs and provided direction for designing HEAs with superior mechanical properties. In the research conducted by Onate et al. [57], the valence electron concentration (VEC) analysis method, computational thermodynamic phase diagram (CALPHAD), and random forest method were employed to predict the doping of Nb in the FeCrMnNiCu system to achieve σ phase stability. Subsequently, detailed analyses of the microstructure were carried out through various characterization methods. By comparing the predicted results of VEC and CALPHAD with the experimental data, the accuracy of these two methods in predicting the stability of HEA phases was verified. This study not only provided new insights into stabilizing the σ phase through Nb doping but also emphasized the importance of combining multiple prediction tools with experimental verification, providing a theoretical basis and technical support for the development of novel high-entropy alloys with specific requirements.

### 2.2. Research Progress on the Regulation of Mechanical Properties of HEAs by Heterogeneous Microstructures

Among all mechanical properties, tensile strength and ductility have the most fundamental connection with practical structural applications and therefore are the most important. Although materials with high strength and excellent ductility are highly sought after, strength and ductility are usually mutually exclusive in most materials, and this is no exception in high-entropy alloys. Through the design of microstructural defects, efforts are being made to resolve this conflict between strength and ductility in high-entropy alloys. Wei et al. [58] conducted in-depth research on the microstructure evolution and dislocation density characterization of the CuNiSiCo alloy. The results show that the tensile strength of this alloy is 937.3 MPa and it has excellent stress relaxation resistance. In the alloy that has undergone cyclic low-temperature rolling and low-temperature aging treatment, there are many dislocation entanglements, dislocation lines, dislocation cells, and deformation twinning. The final average dislocation density is approximately 35.967 ± 1.513 × 10^14^ m^−2^. A strategy to break the strength–ductility trade-off in complex alloy systems was proposed by Professor Yong Liu from Central South University and Professor C.T. Liu from City University of Hong Kong et al. [10] through the controlled introduction of high-density ductile multi-component intermetallic compound nanoparticles. Unlike the brittleness caused by intermetallic compounds in traditional views, this multi-component intermetallic compound nanoparticle-reinforced alloy exhibits an outstanding strength of 1.5 GPa and a tensile elongation of up to 50% at ambient temperatures. Plastic instability is a major issue for high-strength materials, and it can be completely eliminated by generating a unique multi-stage work hardening behavior, which is caused by significant dislocation activity and deformation-induced microbands. Li et al. [59] combined the optimal properties of steel and high-entropy alloys to develop a new type of phase transformation-induced plasticity-assisted biphasic high-entropy alloy. In the phase transformation-induced plasticity-assisted biphasic high-entropy alloy, these two contributions, respectively, lead to enhanced intergranular and interfacial slip resistance, thereby increasing the strength. Furthermore, the increased strain hardening capacity resulted from the dislocation hardening of the stable phase and the phase transformation-induced hardening of the metastable phase, thereby enhancing the ductility. Liang et al. [5] proposed a strategy for designing super-strong high-entropy alloys with high content of nano-precipitates through phase separation. This strategy can simultaneously form strengthening phases and generate a matrix close to the atomic ratio in situ. Moreover, amplitude decomposition was utilized to create coherent nanostructures with low misfit, combining the nearly atomic ordered face-centered cubic matrix with the highly abundant ductile Ni_3_Al-type ordered nano-precipitates. The ultimate alloy strength was close to 1.9 GPa, while maintaining good ductility (>9%). Fan et al. [60] proposed the development of a new type of nano-layered high-entropy alloy with an in-situ formed coherent nano-layered structure. This structure combines unprecedented ultra-high strength and tensile ductility and can be produced through traditional casting and thermal mechanical processing. Studies have shown that significant enhanced tensile ductility can be achieved in the coherent nano-layered alloy, which exhibits a yield strength of over 2 GPa and a uniform tensile elongation of 16%. The ultra-high strength mainly comes from the layer boundary strengthening, while the large ductility is related to the progressive work hardening mechanism regulated by the unique nano-layered structure.

As the research on high-entropy alloys progresses, in addition to adjusting the alloy composition, more effective methods for performance optimization have been proposed. Professor Lu Zhaoping and Professor Wu Yuan from Beijing University of Science and Technology [61] used a TiZrHfNb high-entropy alloy with an equal atomic ratio as the model alloy and added an appropriate amount of oxygen (O). They discovered that the interstitial atoms existed in the high-entropy alloy as ordered interstitial atomic complexes, which was a new state of interstitial atoms between the conventional random interstitial atoms and the ceramic phase. This state could significantly enhance the strength and plasticity of the alloy. This result laid the foundation for designing high-strength and high-toughness refractory high-entropy alloys and promoting their practical engineering applications. It also provided a new idea for solving the contradiction between strength and plasticity in body-centered cubic structure high-entropy alloys. Professor Zhao Yonghao from Nanjing University of Science and Technology and Professor Lu Yiping from Dalian University of Technology [62] used the traditional casting process to prepare a face-centered cubic (FCC)/body-centered cubic (BCC) modulated layered structure AlCoCrFeNi_2.1_ eutectic high-entropy alloy. They achieved an ultimate tensile strength of approximately 1351 MPa and an elongation of approximately 15.4%. Professor Zhang Yong from Beijing University of Science and Technology [63] successfully fabricated Al_0.3_CoCrFeNi high-entropy alloy fibers with diameters ranging from 1 to 3.15 mm using the hot drawing method. In the tensile test of the high-entropy alloy fibers, the precipitated particles and fine grains jointly promoted the continuous high strength and ductility of the fibers. Particularly, the fiber with a diameter of 1.00 mm exhibited significant tensile strength and ductility at 298 K, which were 1207 MPa and 7.8%, respectively. At 77 K, these values increased to 1600 MPa and 17.5%, respectively. Compared with the planar slip deformation mechanism at 298 K, nano-twinned structures induced by deformation appeared after deformation at 77 K, thereby enhancing the tensile strength and ductility at 77 K. Professor Wang Jincheng and Professor Wang Zhijun from Northwestern Polytechnical University [64] proposed a unique concept of phase selection recrystallization, which can fully stimulate the inherent excellent work hardening ability of biphasic alloys and significantly enhance their ductility. Taking the FCC–B2 eutectic high-entropy alloy as the model, by regulating the distribution of the two corresponding strains and the recrystallization behavior, a phase selection recrystallization structure with completely recrystallized soft inclusions embedded in the unrecrystallized framework-like hard phase was obtained. This structure eliminates potential crack sources during deformation, promotes the alloy to undergo continuous work hardening, and enables it to have a uniform elongation rate of up to 35% and a fracture true stress of nearly 2 GPa. On this basis, introducing traditional strengthening mechanisms can further enhance the alloy’s performance. This process has great potential for realizing the application of traditional biphasic alloys as high-strength materials. Professor Li Zhiming from Central South University [65] designed a FeCoNiTaAl multi-component alloy, with the matrix being ferromagnetic and the nanoparticles being paramagnetic (particle size approximately 91 nm, volume fraction approximately 55%). These nanoparticles hindered the movement of dislocations and enhanced strength and ductility. Additionally, their grain size was small, and they had low coherent stress with the matrix. Moreover, their small static magnetic energy formed an interaction volume lower than the width of the magnetic domain wall, minimizing domain wall pinning and thus maintaining the soft magnetic properties. The tensile strength of the alloy was 1336 MPa, the elongation was 54%, the coercivity was 78 A m^−1^, the saturation magnetization was 100 A m^2^ kg^−1^, and the resistivity was 103 μΩ cm. Based on the alloy design strategy proposed in this paper, the high-entropy alloy prepared has perfectly achieved a good combination of high strength, high plasticity, and low coercivity. This work will contribute to the further deep application of soft magnetic materials.

The effective methods reported so far for balancing the strength and ductility in high-entropy alloys include inducing plasticity through stress-induced phase transformation, inducing plasticity through twinning, creating specially tailored eutectic structures, introducing ordered oxygen complexes with vacancies, and generating nano-scale precipitated phases or custom non-uniform structures [9,16,31,61,66,67,68,69]. The strength of high-entropy alloys, like that of other polycrystalline metals and alloys, comes from the inherent lattice resistance to dislocation motion, as well as from various incremental strengthening mechanisms. The yield strength of high-entropy alloys is the sum of lattice friction stress and all other strengthening effects, including grain boundary strengthening, precipitation strengthening, twin boundary strengthening, and phase transformation-induced strengthening [66]. Lattice friction force, solid solution strengthening, dislocation strengthening, and grain boundary strengthening usually exist in all polycrystalline alloys, while precipitation strengthening, twinning strengthening, and phase transformation-dominated strengthening only occur in certain specially designed alloys [66]. These strengthening mechanisms are not necessarily present in all high-entropy alloys. We can selectively control the proportion of each mechanism in the alloy through different processing methods and the selection of component combinations, in order to achieve the targeted control of the alloy’s properties. However, the above preparation methods and control means are relatively complex and costly. To further promote the industrialization development of high-entropy alloys, it is urgent to find effective multi-type microstructure defect collaborative control methods and deeply explore the collaborative deformation mechanism of multi-type microstructures.

### 2.3. Research Progress on Heterogeneous Microstructure AM-HEAs

Additive manufacturing technology is a revolutionary rapid prototyping technique, which is a layer-by-layer manufacturing process of materials through digital assembly that rapidly converts a virtual three-dimensional model into a tangible object, without being limited by traditional casting, forging, and processing procedures [70,71,72,73]. The development of additive manufacturing technology as a near-net-shape forming process provides an effective method for manufacturing components with complex and specific shapes. This technology can conveniently design and print complex parts composed of multiple components, thus achieving great design freedom [18,70].

Currently, according to the ISO/ASTM standards, additive manufacturing technologies can be classified into seven major categories, namely binder jetting, directed energy deposition, material extrusion, material jetting, powder bed fusion, sheet lamination, and vat photopolymerization. However, the most popular additive manufacturing technologies for metals are mainly directed energy deposition (DED) and laser powder bed fusion (LPBF), while the materials used in the other additive manufacturing technologies are mostly plastics, waxy materials, photopolymer materials or resins, etc. [74].

Directed energy deposition (DED) and laser powder bed fusion (LPBF) each have their unique advantages. For instance, DED is suitable for repairing existing components or adding complex features to existing parts. It can precisely deposit materials in specific areas without the need for reconstruction, and DED typically deposits materials at a faster rate, which is particularly beneficial for rapid prototyping and production. By adjusting process parameters, DED can create gradient materials with gradually changing compositions, thereby optimizing the performance of parts. In contrast, LPBF technology can achieve very high resolution and is suitable for manufacturing parts with complex internal structures and fine external details, such as medical implants and aerospace components. By precisely controlling the melting process of each layer, parts produced by PBF often exhibit excellent mechanical properties, including high strength and good ductility, and the surface quality of PBF-produced parts is relatively good, reducing the need for subsequent processing. These characteristics make these two additive manufacturing technologies outstanding in manufacturing complex and high-performance metal parts. Professor Lu Yiping’s team from Dalian University of Technology [75] successfully prepared bulk kilogram-scale samples of eutectic high-entropy alloys with layered structures using additive manufacturing technology, providing a new approach for optimizing the performance of high-entropy alloys. The printed AlCoCrFeNi_2.1_ eutectic high-entropy alloy samples exhibited excellent plasticity and ultra-high strength, with a tensile yield strength of 1050 MPa and a tensile elongation of nearly 24%, along with a density of 99.998% and excellent seawater corrosion resistance. Professor Liu Zhiyuan from the Institute of Additive Manufacturing at Shenzhen University and Professor Jia Zhe from Southeast University [76] collaborated to prepare (FeCoNi)_86_Al_7_Ti_7_ precipitation-strengthened high-entropy alloys using selective laser melting technology and studied the effects of different temperature aging heat treatments on the strength and plasticity of the precipitates in the high-entropy alloys. This research not only successfully explained the strengthening mechanism of additive manufacturing precipitation-strengthened high-entropy alloys but also provided theoretical references for the plastic deformation and fracture processes of additive manufacturing metal materials. Guan, S. et al. used laser net shaping technology to prepare equiatomic CrMnFeCoNi high-entropy alloys, which formed microstructures at multiple scales, including columnar grains, solidification substructures, and dislocation substructures. The tensile yield strength of the directly formed alloy was comparable to that of the finer-grained forged and annealed alloy [77]. Sun et al. directly printed a CoCrNiFeMn high-entropy alloy with multi-level heterogeneous microstructures such as columnar grains and ultrafine dislocation cell-like structures using LPBF. This sample still exhibited excellent thermal stability after long-term exposure to high temperatures, with minimal hardness reduction and no obvious recrystallization phenomenon. This is in sharp contrast to the significant performance decline of traditional processed high-entropy alloys after exposure to high temperatures [78].

The research on data-driven high-entropy alloy prediction technology mentioned earlier has also played an important role in the study of additive manufacturing of high-performance high-entropy alloys. Cagirici et al. [79] used thermophysical calculation methods and CALPHAD to predict the phase formation and stability of high-entropy alloys during electron beam melting. Through basic thermophysical calculations, they determined that adding Ti is conducive to the thermodynamic stability formation of secondary phases, such as σ phase and Cr-rich γ phase. Based on CALPHAD simulation, they selected three different Ti contents of HEAs for preparation. In the EBM process, they exhibited the expected microstructure characteristics and mechanical properties. This study shows that by adjusting the Ti content, the microstructure of HEAs can be controlled during the EBM process, and the mechanical properties can be optimized. Shah et al. studied the use of physical and computer simulation to predict the microstructure evolution of eutectic high-entropy alloys under the PBF method [80]. This study used the Rosenthal equation to estimate the temperature distribution under welding conditions and used the phase field simulation method to simulate the microstructure evolution of alloys under different processing conditions. Through BSE-SEM analysis of samples, it was shown that with the increase in the number of thermal cycles, the element distribution within the σ phase changed, which proved that the phase field simulation can effectively predict the microstructure evolution under AM conditions. Liu et al. studied the microstructure and properties of AlxCoCrFeNi high-entropy alloys prepared by SLM [81]. They conducted in-depth discussions through molecular dynamics simulation and other means. The simulation process was divided into the relaxation stage, the laser heating stage, and the rapid solidification stage. Through this approach, the behavior of the material during SLM was analyzed in detail. This study helps to reveal the intrinsic influencing mechanism of microstructure formation under non-equilibrium solidification conditions in SLM, especially the changes in columnar crystal growth rate, morphology, stress distribution at grain boundaries, and defect structures under different Al concentrations. The study also indicates that substrate temperature can improve the solidification formation performance and reduce microstructure defects, and the fluctuation of Al concentration inhibits the high cooling rate caused by steep temperature gradients. This is of great significance for designing SLM-prepared HEAs with specific microstructures and excellent properties. Zhou et al. developed a deep learning algorithm that depends on additive manufacturing process parameters and data augmentation [82]. They compared the performance differences between DL models and ML models on the test set using a confusion matrix. They trained the DL model using nine phase types and utilized this new phase classification method to enable the model to predict the phase structure formed under specific conditions based on these features. After integrating AM parameters and performing data augmentation in the DL model, the prediction accuracy of the DL model increased to 91.23%. This study not only demonstrates how to improve the prediction accuracy of the multi-phase structure of additive manufacturing high-entropy alloys through DL and data augmentation techniques, but also proves the practicality of the proposed method, providing data support for further in-depth research on the phase prediction of additive manufacturing high-entropy alloys with strong data guidance.

A variety of research results have demonstrated that various data-based learning or computing models play a significant role in studying the microstructure of high-entropy alloys in additive manufacturing, opening up a new path for additive manufacturing of high-entropy alloys. By studying the complex multi-component parameters of high-entropy alloys and the parameters of additive manufacturing as feature data, and verifying and calibrating the model learning results through a small number of experiments, the massive experiments are transformed into ultra-large datasets that require high computing power to process. Finally, a model based on experimental data reinforcement is used to predict the microstructure obtained from the additive manufacturing of unknown component high-entropy alloys, thereby reducing the time cost required for massive experiments and simplifying the exploration process for the components used in additive manufacturing of high-entropy alloys. Moreover, by taking additive manufacturing parameters as one of the features, a certain type of manufacturing parameter with good performance can be independently summarized. This has guiding significance for the exploration of parameters for other materials used in additive manufacturing.

By reducing material waste and eliminating processing steps, additive manufacturing technology shows great potential in reducing energy consumption during the manufacturing process, which is almost impossible to achieve in traditional manufacturing. This processing method not only brings breakthrough performance that traditional processes cannot achieve, and the flexibility brought by the operation process, but also plays a special role in various industries. In particular, laser additive manufacturing technology, with its significant temperature gradient and high cooling rate, has distinct characteristics that are markedly different from traditional manufacturing techniques. It not only leads to micro-defect structures such as fine grains or non-uniform grains and sub-micron-nanometer-level subgrains, but more importantly, due to the characteristics of thermal cycling, it generates high-density dislocation network defect structures [83,84]. The development of this technology offers the possibility for the development of new high-entropy alloy materials and efficient control of microstructure configuration, and holds significant technical value and theoretical guiding significance.

## 3. Constructing Heterogeneous Microstructures in High-Entropy Alloys Through Additive Manufacturing Processes

### 3.1. Different Types of Heterogeneous Microstructures

**Figure 1:**
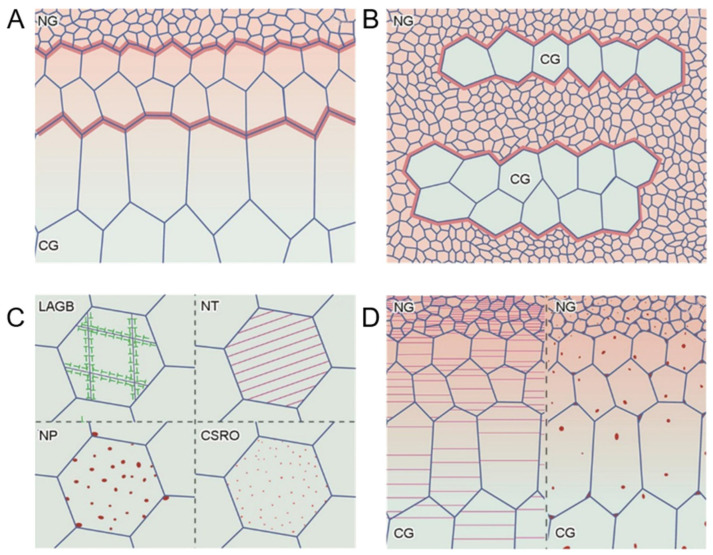
Typical heterostructure schematic diagram: (**A**) gradient structure, (**B**) layered structure, (**C**) various heterostructures including low-angle grain boundaries, nanotwins, nano-precipitates, and chemical short-range order, (**D**) mixture of multiple heterostructures. The figure is adapted from the literature [85].

The microstructure usually refers to the regions within a material that have different compositions, phases, or organizational structures at the microscopic scale. This heterogeneity can be observed at the nanoscale or micrometer scale and can significantly affect the overall properties of the material, such as mechanical properties, electrical properties, magnetic properties, thermal properties, etc. Typical heterogeneous microstructures include gradient structures, layered structures (or heterogeneous layered structures), nano-precipitates, and various chemically short-range ordered structures. Figure 1 shows the schematic diagrams of various heterogeneous microstructures, illustrating the superposition of gradient structures, layered structures, and multiple heterogeneous structures [85]. The traditional methods for preparing heterostructures mainly include surface mechanical rolling treatment (SMAT), rolling and annealing (RA), and accumulative roll bonding (ARB). SMAT is a surface treatment technology. The principle involves enhancing the mechanical properties of the surface layer through the generation of high-density dislocations and grain refinement at the material surface. The process involves using hard materials to impact the surface of the material at high speed. Through causing plastic deformation of the surface layer, a nano-scale grain structure is formed. When the surface is impacted, as the distance from the surface increases, the impact energy received by the structure decreases. Therefore, the areas closer to the surface will experience more intense plastic deformation and more accumulation of dislocations, resulting in a more significant refinement of the grain structure. Thus, SMAT is mainly used for preparing gradient structures. RA achieves a reduction in material thickness through a series of rolling steps and then undergoes annealing after one or several steps to eliminate internal stress and restore the ductility of the material. This method not only changes the size and shape of the material, but also optimizes the microstructure by adjusting parameters, and can be used for processing heterogeneous grain structures. ARB is a special rolling technique used to manufacture ultra-fine-grained or nanocrystalline materials. This process typically involves overlapping two or more metal sheets for cold rolling, and then repeatedly folding and rolling the resulting composite material. This process is repeated multiple times, and each rolling process makes the material thinner and causes intense plastic deformation between the layers, resulting in finer grain size. The ARB method can make it easier to prepare heterogeneous layered structures.

The gradient structure refers to a structure within a material where the composition, microstructure, or properties gradually change in one direction. This structure can be achieved through processes, such as using surface treatment technology (SMART), to gradually reduce the hardness from the surface to the interior of the material, thereby enhancing its wear resistance and fatigue performance. In AM, gradient compositions can be prepared in HEAs by controlling the filament material [86]. The layered structure consists of multiple thin layers of different properties which are treated differently. This structure can effectively improve the material’s fracture toughness, wear resistance, etc. Low-angle grain boundaries are defined as grain boundaries where the orientation difference between two parts is less than 10–15°, composed of a series of dislocations. Twinning refers to a situation where one part of a crystal is symmetrically mirror-imaged relative to another part through a specific plane. When the thickness of these twin crystals reaches the nanometer scale, they form nanotwinning. Nanoprecipitates refer to second-phase particles of nanoscale size distributed in the matrix. These nanoprecipitates can effectively hinder dislocation movement and enhance material strength. Chemical short-range order refers to the phenomenon in alloys where certain atoms tend to locally aggregate rather than randomly distribute. This phenomenon also affects various properties of the material to a certain extent.

The above briefly introduced the traditional methods for preparing heterostructures. Compared with these methods, AM has inherent advantages in the preparation of heterostructures. For instance, AM technology, through the sequential deposition of materials layer by layer, can easily construct gradient structure materials that transition from one material to another. The laser powder bed fusion technology can use different powders in the same construction process, thereby directly manufacturing components with heterostructures.

For example, during the DED process of preparing HEAs, an interlayer pause strategy was adopted, resulting in the preparation of an AlCoCrFeNi_2.1_ sample with an alternating nanoscale layered structure. The eutectic layers of this sample were 40% more refined compared to the situation without the interlayer pause strategy, and their strength and ductility increased by 14% and 7%, respectively [87]. The temperature gradients and cooling rates at different positions during the DED process also led to the emergence of various hetero-microstructures. Qingxuan Sui et al. prepared CoCrFeNiMo samples with algal-like eutectic structures, zigzag-layered structures, and irregular layered structures at the top, middle, and bottom, respectively [88]. The fine eutectic layer structures caused the samples to have high hardness and high wear resistance [88]. This characteristic of being able to directly prepare samples with hetero-microstructures by changing the printing strategy or utilizing the inherent characteristics of the printing method makes additive manufacturing technology an attractive preparation method.

Table 1 is a summary of the properties, compositions, microstructures, and corresponding additive manufacturing technologies of the high-entropy alloy samples mentioned in the manuscript.

### 3.2. Additive Manufacturing of Gradient-Structured High-Entropy Alloys

The gradient structure refers to a special design of a material where its composition, microstructure, or properties gradually evolve along a specific direction within the material itself. This results in the material properties (such as hardness, elastic modulus, thermal expansion coefficient, etc.) changing continuously or gradually in that direction in order to meet specific engineering requirements, such as components operating in extreme environments, or for manufacturing human implants. The source of the gradient can be changes in composition, changes in phase composition, changes in grain size, or the presence of various defect aggregations.

HEA, as an emerging material with excellent performance, combines the gradient structure with HEAs and realizes it through AM technology [86,94,95]. The aim is to develop a new generation of engineering materials with outstanding performance through innovative design concepts and technical means. The figure shows the total cross-sectional view, corresponding local magnification view, construction direction diagram, XRD spectrum and lattice parameter diagram of the functional gradient AlxCoCrFeNi HEAs with varying aluminum concentration along the construction direction, which are fabricated in situ using additive manufacturing [86].

**Figure 2:**
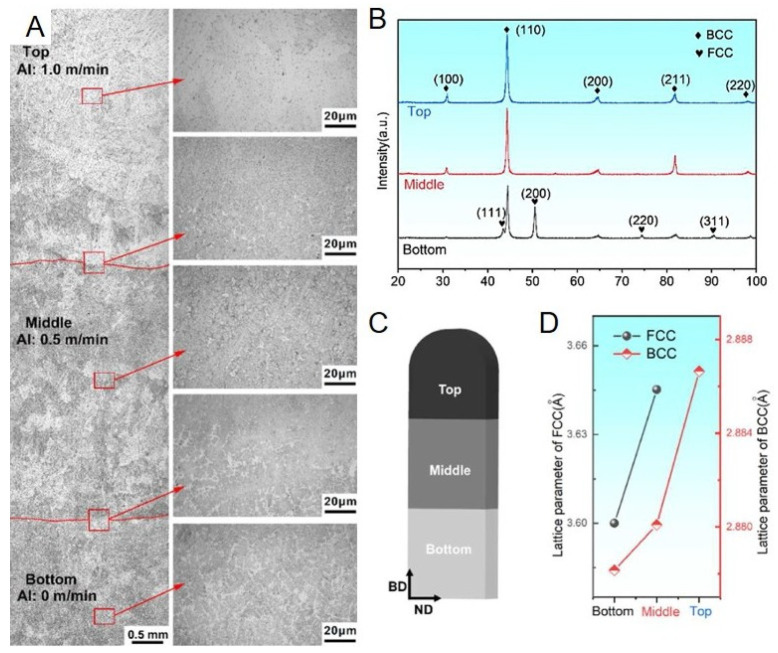
(**A**) Total cross-sectional images and local magnification images of AM-HEA samples, (**B**) XRD spectra, (**C**) schematic diagram of the construction direction, (**D**) lattice parameters of FCC and BCC phases as the aluminum content increases, adapted from reference [86].

This sample was fabricated through wire arc additive manufacturing (WAAM) technology based on the welding principle, using a composite core wire (consisting of two aluminum wires, two nickel wires, one cobalt wire, one iron wire and one 304 stainless steel wire, among which 304 contains approximately 68 atomic percent of iron, 21 atomic percent of chromium and 11 atomic percent of nickel) as the raw material. Figure 2A shows a smooth transition from low-aluminum alloy to high-aluminum alloy, and Figure 2B indicates that the top of the sample is a BCC phase, while the bottom is a dual-phase solid solution structure composed of BCC and FCC phases. This phenomenon can be attributed to the relatively larger atomic radius of aluminum atoms. As the construction direction increases, the concentration of them increases, causing a gradient change in the lattice distortion, promoting the transformation of the FCC lattice to a denser BCC lattice. This change allows for the accommodation of more aluminum atoms. The lattice constant changes shown in Figure 2D also confirm this change. The lattice constant of the FCC phase gradually approaches and eventually disappears towards the BCC phase. These figures collectively demonstrate that as the aluminum content changes along the construction direction, the phase composition also changes, forming a continuous transition from the bottom’s FCC–BCC dual-phase structure to the top’s BCC single-phase structure. This is a typical gradient structure where the phase changes gradually with the composition.

**Figure 3:**
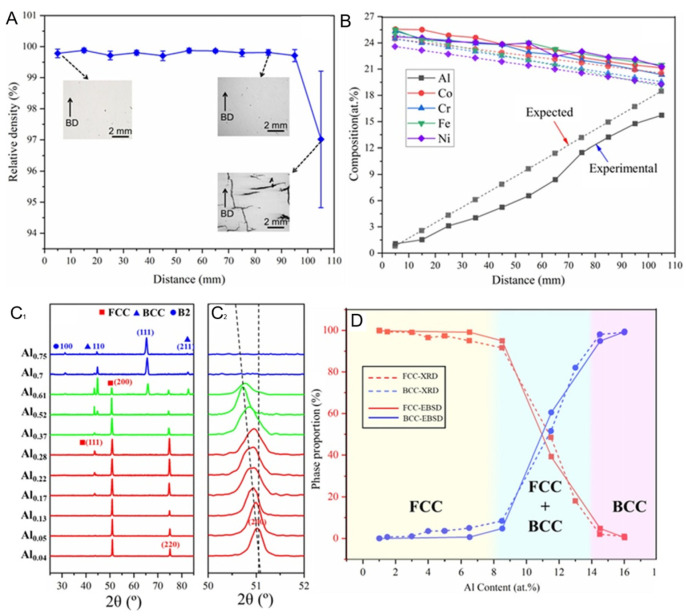
(**A**) The relative density of AlxCoCrFeNi high-entropy alloys along the GD direction, (**B**) distribution of element, (**C1**) XRD diffraction patterns of AlxCoCrFeNi gradient heterogeneous alloys in eleven regions, (**C2**) the shift in the FCC (200) peak position is indicated by the dotted line. The inclined dotted line represents the shifted peak position, while the vertical dotted line represents the original peak position before the shift. (**D**) The variation in phase content with Al content was calculated through MAUD fitting (dashed line) and EBSD statistics (solid line). Adapted from reference [89].

Furthermore, Guo et al. utilized a custom LPBF device and used AlCoCrFeNi and CoCrFeNi powders in an equal atomic ratio as raw materials to prepare AlxCoCrFeNi-HEAs with compositional gradients, aiming to identify the composition range that enables crack-free manufacturing [89]. In the experiment, a self-designed powder supply hopper was used to achieve continuous gradient manufacturing, allowing the composition to change continuously along the construction direction. The element distribution is shown in Figure 3B. The chemical composition shows a gradient change, and the change is smooth. It is mainly identified based on the Al content in the samples, and the quantitative phase composition of the gradient alloy was obtained using the MAUD software (version 2.9997).

The figure above shows that both FCC and BCC phases were observed throughout the range of Al content (0.04–0.75). Except for the Al 16 at% composition, no obvious cracks were observed in the samples prepared with the other compositions. The gradual microstructure was classified into three categories based on the fraction of phases: FCC dominant, BCC dominant (phase fraction > 90%), and mixed phase. When x was within the range of 0.37–0.61, the proportion of BCC phase increased rapidly. When the Al content further increased, the BCC phase began to dominate. It was noted that the ordered B2 phase was only observed in the composition region where BCC dominated (x = 0.61–0.75). It was shown in the enlarged image near the (200) peak of FCC (Figure 3(C2)) that 2θ continuously shifted to lower angles with the increase in Al content, indicating that the increase in Al content induced lattice parameter expansion. This result is exactly the same as that observed in the previous study.

The gradient structures prepared by the above two studies are completely based on modifying the AM parameters to directly prepare high-entropy alloys with gradient structures that change with composition. By creating composition gradients within the same sample to screen out the compositions that are not suitable for forming, this reduces the waste of powder to a certain extent. This is particularly important for expensive high-entropy alloys, which helps to reduce costs. The performance comparison between different test samples obtained within the same block is more intuitive, eliminating the reason for the mechanical property differences in the samples caused by external factors during the preparation process.

### 3.3. Additive Manufacturing of Heterogeneous Layered Structure High-Entropy Alloys

**Figure 4:**
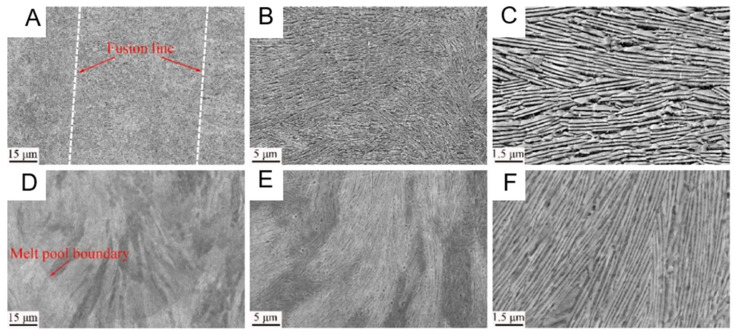
SEM images of the Ni_2.1_ sample prepared by SLM: (**A**–**C**) perpendicular to the construction direction, (**D**–**F**) along the construction direction; (**B**,**C**,**E**,**F**) are high-magnification SEM images of the eutectic structure, adapted from reference [90].

Heterogeneous layered structures usually refer to composite structures composed of different types of materials or layers with different crystal structures, chemical compositions, etc. These layers are combined together through physical or chemical methods to form a whole, but each layer still retains its unique physical and chemical properties, thereby enhancing the various properties of the material. Figure 4 shows the microstructure of oriented-grown ultrafine eutectic lamellar with a directional growth [90]. The AlCoCrFeNi_2.1_ eutectic high-entropy alloy was prepared by selective laser melting (SLM) technology; Figure 4A–C are SEM images perpendicular to the construction direction, and Figure 4D–F are images along the construction direction. Figure 4B,C,E,F are high-magnification SEM images of the eutectic microstructure. The fine lamellae are B2 phase, and the coarse lamellae are FCC phase. During the laser additive manufacturing process, the Ni_2.1_ eutectic alloy can form a cellular structure, and as the printing parameters change, the layered structure will evolve into a cellular structure. In SLM, the chemical composition, liquid phase temperature gradient G, and solidification rate R will determine the final formed structure morphology through influencing the solidification mode of the eutectic structure [90]. When G is small and R is large, the fine grains formed at the solidification interface will continue to grow due to the instability of the crystal interface, thereby continuing to increase the temperature gradient and concentration gradient in the overcooled liquid phase, leading to compositional undercooling and the formation of cellular crystals or cellular dendrites.

In Figure 4A,D, the scanning trajectory of the laser and the molten pool structure formed during the printing process are shown. This scanning trajectory is an inherent feature of laser additive manufacturing. Meanwhile, the width of the scanning trajectory shown in Figure 4A is approximately 71 μm, which is consistent with the preset value [90]. Figure 4B–E further magnifies and displays the layered eutectic structure. The growth direction of the layered microstructure of this sample shows a high degree of regularity, and its growth direction is perpendicular to the contour of the molten pool. This is because, during the printing process, the growth direction of the eutectic structure is more inclined to be consistent with the maximum heat flow direction.

Compared with the lamellar spacing of the as-cast Ni_2.1_ (about 3.5 μm) [96], the samples prepared by SLM have a smaller lamellar spacing (about 211 nm). This is because the cooling rate in the SLM process is higher, which inhibits the growth of the two phases. Meanwhile, the eutectic structure grows in the -direction perpendicular to the contour of the molten pool because the direction of the maximum heat flow affects the direction of the eutectic structure growth.

**Figure 5:**
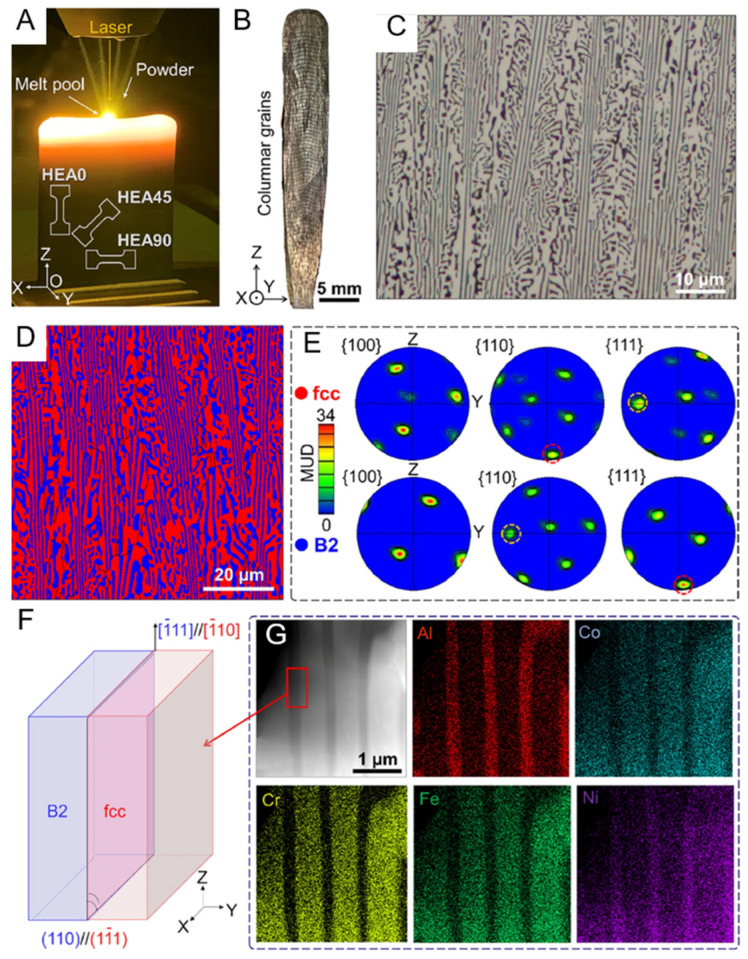
(**A**) Illustration of sample plate preparation by DED method and the position for stretching sample collection; (**B**) illustration of the construction direction of DED-HEA and the macroscopic pattern; (**C**) directional co-crystalline optical micrographs of ultrafine lamellae and branched eutectic cells; (**D**) EBSE phase diagram showing the directional co-crystalline FCC and B2 phases; (**E**) corresponding pole figures of FCC and B2 phases, showing the K–S orientation relationship between the two phases and related MUD; (**F**) illustration of the K–S orientation relationship between FCC and B2; (**G**) EDS spectra obtained by TEM, showing the segregation of Al and Ni to the B2 phase and the segregation of Co, Cr, and Fe to the FCC phase, adapted from reference [92].

The directed energy deposition (DED) method has been used to fabricate directional high-entropy alloy materials consisting of FCC and B2 phases in ultrafine eutectic layer sheets. The AlCoCrFeNi_2.1_ prepared by DED shows a directional solidification microstructure with continuously growing columnar grains along the construction direction [92]. The construction direction is shown in Figure 5B. The microstructures present in Figure 5C,D indicate that the sample is composed of FCC and B2 eutectic grains. Figure 5E shows that the {110} pole figure of the FCC phase and the {111} pole figure of the B2 phase have the maximum uniform density (MUD) value in the construction direction, which indicates that the preferred growth directions of FCC and B2 are <110> and <111>, respectively. Additionally, the {111} pole figure of FCC and the {110} pole figure of B2 have a common uniform density multiple MUD point in the Y direction, indicating that the orientation relationship between the FCC phase and the B2 phase is K–S type. This matching relationship makes the atomic arrangement at the phase interface more coordinated, which is conducive to the transfer of dislocations and stress distribution during subsequent deformation.

The rapid cooling of DED may have inhibited certain phase transformation paths, such as the formation of other possible ordered phases (such as L12 or L10 phases), thereby resulting in a stable FCC–B2 eutectic structure. Additionally, it was observed in the paper that the martensitic phase transformation (the stress-induced strain of the B2 phase during stretching) is also constrained by the initial crystallographic orientation formed by DED.

During the DED process, the local energy input from the laser beam will generate significant temperature gradients in the material. Additionally, as mentioned earlier, DED has a high cooling rate. The combination of these two factors makes it easier to form directional microstructures. In the molten pool within the laser-heated area, the solidification front moves very quickly. This rapid solidification promotes the emergence of the directional nanosheet structure formed through epitaxial growth. At the same time, DED allows for the control of the type and quantity of materials added in each layer, indicating that DED technology enables the adjustment of the chemical composition between different layers, thereby facilitating the formation of heterogeneous layered structures in two-phase or multi-phase material systems.

### 3.4. Additive Manufacturing of Multi-Level Heterogeneous Structure High-Entropy Alloy

**Figure 6:**
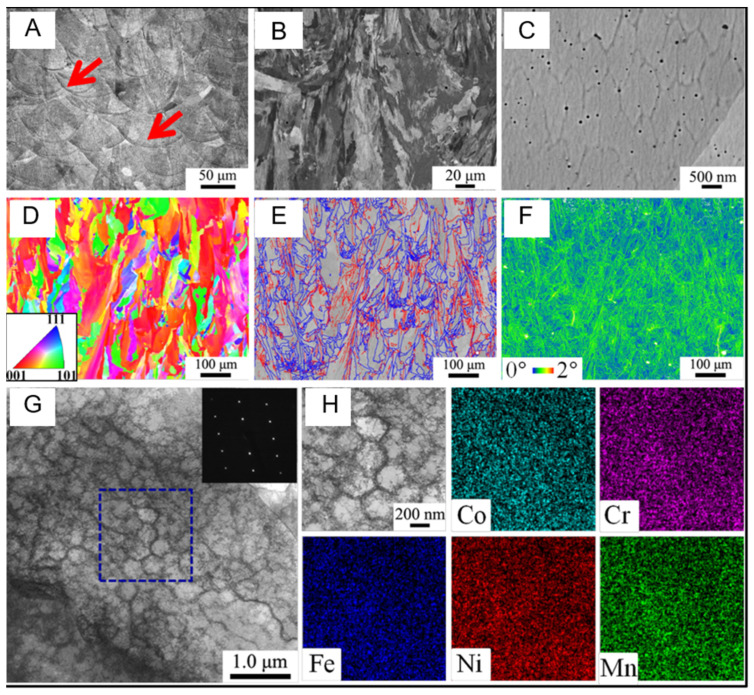
(**A**) Frontal optical microscopic image, (**B**) SEM image, (**C**) SEM image of honeycomb structure, (**D**) frontal electron EBSD-IPF map, (**E**) EBSD image quality map: the blue line represents HAGBs (high-angle grain boundaries), the red line represents LAGBs (low-angle grain boundaries). (**F**) Frontal lattice mismatch degree (KAM) image, (**G**) bright-field scanning transmission electron microscopic image of the honeycomb structure and its corresponding selected area electron diffraction (SAED) pattern, (**H**) the bright-field STEM image of the area marked by the box in (**G**) and the element distribution map of this area, adapted from reference [91].

Multi-level heterogeneous structures usually refer to multiple different scales and types of microstructures existing simultaneously in a single material [91,97]. The core features of these structures are multi-level nesting and heterogeneity. Secondly, due to the diversity of the arrangement and combination of various microstructures, the composition of multi-level heterogeneous structures exhibits high flexibility. Through the coordinated action of different scales and structures, the material exhibits specific mechanical properties. As shown in Figure 6, a dense CoCrFeNiMn multi-entropy alloy was prepared by selective laser melting [91].

The characterization results show that the multi-level heterogeneous structure is composed of four scales of organization, namely the macroscopic molten pool structure (Figure 6A), the micro-level columnar grains (Figure 6B), the epitaxial growth of the grains, the sub-microscopic level sub-micron cellular structure (Figure 6G,H), and the nanoscale dislocation grid. Referencing the similar structure in the manufacturing of 316L stainless steel [98], the cell walls are covered with high-density dislocations. The energy spectrum analysis in Figure 6H indicates that the element distribution of the sample is uniform, with no obvious segregation, suggesting that the rapid cooling during the printing process inhibited the segregation process, resulting in the final prepared sample exhibiting a typical multi-level heterogeneous structure combination.

**Figure 7:**
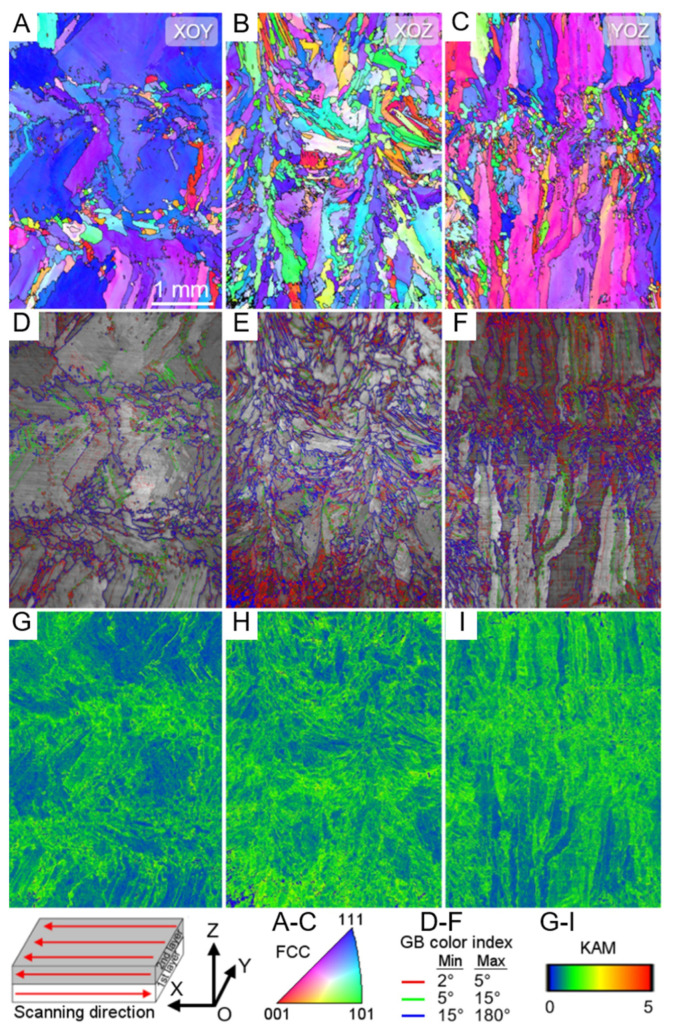
The EBSD results of LMD-deposited iHEA-Nb samples (**A**–**C**) plotted relative to the stretching direction, (**D**–**F**) grain boundary map, and (**G**–**I**) KAM map, adapted from reference [93].

Another study used laser cladding deposition (LMD) technology to synthesize an Fe_49.5_Mn_30_Co_10_-Cr_10_C_0.5_ (iHEA-Nb) interstitial solid solution high-entropy alloy containing 0.5% Nb in situ. Multi-level heterogeneous structures were designed in the prepared samples, including heterogeneous grain structures, cellular substructures, precipitated phases, and element segregation [93]. Figure 6 shows that the sample has a typical heterogeneous grain structure, including large and long columnar grains and equiaxed grains. Continuous nucleation of equiaxed fine grains was detected around the laser trajectory and layer interface, indicating that there are significant differences in grain sizes at different positions. The overall KAM results in Figure 7G,H show that there is a higher strain level near the grain boundaries, suggesting a large accumulation of dislocations. In addition, the presence of Nb element leads to severe lattice distortion, promoting solid solution strengthening and increasing the composition overcooling of the liquid phase during solidification, thereby forming more fine grains. These sub-grain boundaries and dislocation aggregation areas collectively reflect the heterogeneity in the microstructure.

**Figure 8:**
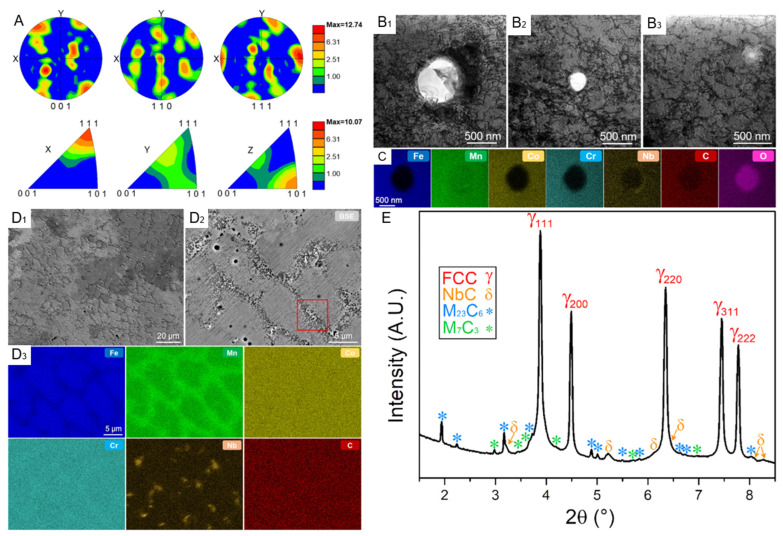
(**A**) PFs in the XOY plane and IPFs in the XOY plane, (**B1**–**B3**) enlarged images of circular precipitated phases of different sizes, (**C**) EDS images of the precipitated phases, (**D1**–**D3**) backscattered electron images and corresponding element distribution maps of the cellular substructures in the iHEA-Nb samples, (**E**) synchrotron X-ray diffraction patterns of the iHEA-Nb samples with vegetation obtained by laser cladding technology, adapted from reference [93].

Figure 8A further presents the texture characteristics of the sample through polar plots and inverse polar plots on the XOY plane. It is noted that iHEA-Nb has the highest MUD in the <111> direction and also exhibits relatively high MUD in the <110> direction. This indicates that crystal orientation is also uneven on a macroscopic scale, further confirming the heterogeneity of the material. Moreover, when the material is stretched along the <111> and <110> orientations, deformation twins are more likely to form. Therefore, the <111> and <110> orientations can better balance strength and ductility [99]. Figure 8(B1–B3) shows the enlarged images of three different-sized circular precipitates existing within iHEA-Nb. Dislocation entanglements are detected around these phases. Figure 8C of EDS indicates that the precipitates are manganese-rich oxides, which may be caused by contamination during the preparation process or insufficient argon protection in the LMD process. Figure 8(D1–D3) shows the cellular substructures existing in the material. The reason for the existence of this substructure is that the cooling rate during laser additive manufacturing is too fast, resulting in a large number of dislocations remaining. In addition, the element distribution in Figure 8(D3) shows that there are component differences in the cellular structure, which is related to the melting point differences between different elements.

Figure 8C presents the XRD spectrum of the sample, showing that in addition to the FCC phase, the sample also contains nano-sized precipitated phases such as NbC, M_23_C_6_, and M_7_C_3_. These nano-scale phase structures further enhance the strength and toughness of the material. The existence of multi-level heterogeneous structures is of vital importance for enhancing the overall performance of materials. Through the coordinated cooperation among various microstructures, the performance of the materials can be further strengthened, which also provides a new idea for the design of high-performance alloys.

## 4. Mechanical Performance Analysis

### 4.1. Additive Manufacturing of Gradient Structure Materials

The gradient structure improves the mechanical properties of materials by creating continuous variations in composition, microstructure, or both within the material. For example, in component gradient multi-entropy alloys, the degree of lattice distortion changes with the variation in solute atom concentration, thereby increasing the hardness and yield strength of the material. The resulting phase composition changes and uneven phase distribution helps alleviate the problem of stress concentration and improves the toughness of the material. During the deformation of gradient structure metals, the mechanical incompatibility between the gradient layer and the coarse grain layer inevitably leads to heterogeneous plastic deformation, resulting in a strain gradient. Subsequently, the microstructure adapts to this strain gradient, generating dislocation walls or dislocation accumulations, providing dislocation strengthening and strain hardening. This mechanism is evident in various gradient materials [86,97].

Lu et al. conducted in-depth research on the mechanical properties of AlxCoCrFeNi GHEA fabricated by dual-wire arc additive manufacturing. They tested the microhardness and compressive properties of different regions of the samples and observed the changes in the microstructure of different regions of the material during the testing process in order to analyze the differences in mechanical properties obtainable in different regions. The accurate data are summarized in Table 2, which presents the hardness of the samples prepared in the work, namely yield strength (YS), fracture strength (FS), and plastic strain (PS).

Along the construction direction, the content of Al gradually increases, while the content of BCC phase also increases. Its characteristic is being hard and brittle. Therefore, an increase in the proportion of BCC phase will enhance the hardness and yield strength, but it will lead to a decrease in fracture strength and plastic strain.

**Figure 9:**
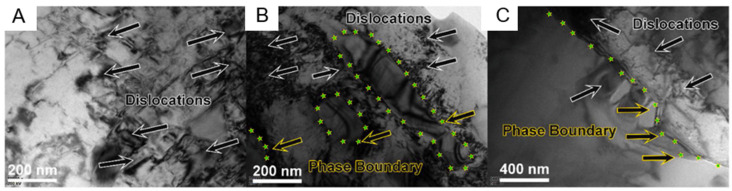
(**A**) Dislocation accumulation and entanglement in the bottom area, (**B**) dislocation accumulation and entanglement in the middle area, (**C**) dislocation accumulation and entanglement in the top area, adapted from reference [86].

In alloys, dislocations are distributed at the phase boundaries and within the grains. When subjected to external stress, the phase boundaries become obstacles for the movement of dislocations, thus causing dislocation accumulation, forming high-density dislocation units, and generating hard microstructures, which act as barriers to prevent further movement of dislocations. When the grain size decreases, the area of grain boundaries increases, and the obstruction effect on dislocation movement also increases. Figure 9A shows the TEM image of the bottom area of the sample. Dislocation accumulation results in a grid-like structure. Therefore, at the bottom of the sample, the strengthening effect mainly comes from dislocations and grain boundary strengthening. As can be seen from Figure 9B,C, the enriched Cr precipitated phase is distributed within the grains, but its effect is not significant. Elemental segregation in the alloy alters grain boundary properties, reducing grain boundary strength and promoting crack initiation and propagation [100]. In the top area, Cr elements are enriched at the grain boundaries, and the grain boundary strength decreases. Therefore, during the testing process, it is prone to produce intergranular fracture, which is an important factor contributing to the reduced plasticity of the top alloy.

**Figure 10:**
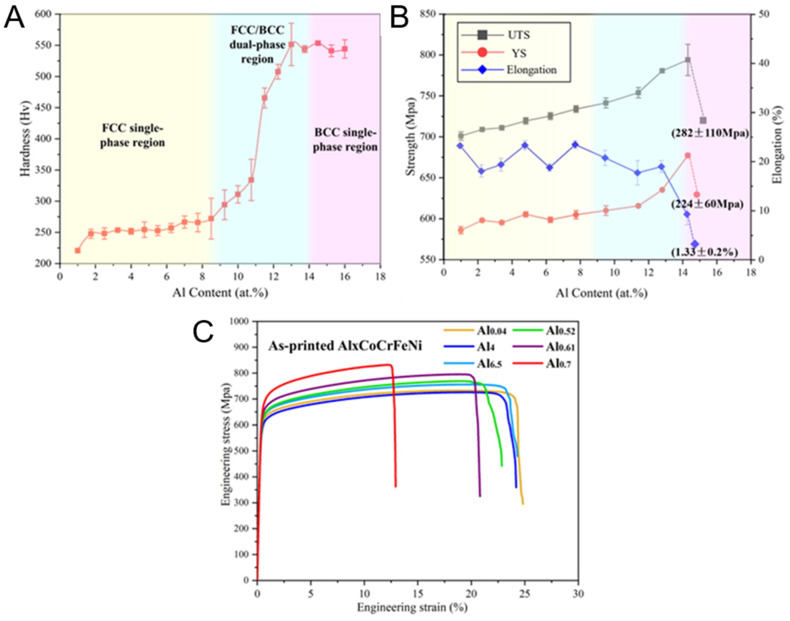
(**A**) Hardness, (**B**) the relationship between alloy mechanical properties and the molar ratio of Al, (**C**) engineering stress–strain curve. Adapted from reference [89].

In the study by Guo et al. [89], the mechanical properties exhibited by the samples changed in a pattern consistent with that of the research by Lu et al. The increase in Al content leads to the emergence of hard and brittle BCC phases, resulting in an increase in overall strength and a decrease in plasticity. The more detailed curves shown in Figure 10 reflect this trend.

In Figure 10A, the hardness rapidly increases within the range of x = 0.37–x = 0.7, which is in good agreement with the observed rapid increase in the BCC phase composition. However, no rapid increase stage was shown during the strength change process. The elongation remained linearly increasing within the Al content variation range. The elongation decreased overall as the phase fraction increased. In this material with both hard and soft phases, plastic deformation usually starts from the soft phase when loaded. As the loading continues, the strain accumulates at the interface between these regions through geometrically necessary dislocations (GND). This structure causes an increase in the material’s hardness, i.e., processing hardening occurs. However, it also generates a local stress field, which becomes a potential crack source. If not handled properly, it may lead to premature failure of the material.

### 4.2. Additive Manufacturing Layered Structural Materials

**Figure 11:**
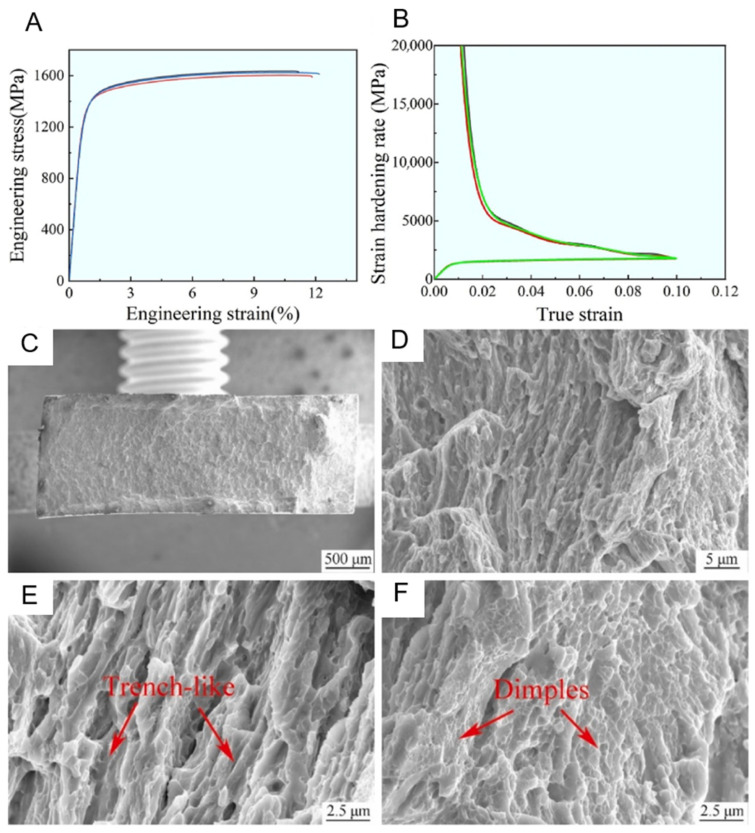
(**A**) Stress–strain curve of the sample prepared by SLM, (**B**) strain hardening rate and true stress–strain curve, (**C**) low-magnification fracture surface morphology of the sample prepared by SLM, (**D**–**F**) high-magnification SEM images showing the groove-like structure and ductile pits, adapted from reference [90].

The main reason why the heterogeneous layered structure can improve the material properties lies in its unique microstructure features, which bring about various strengthening mechanisms, such as the strengthening effect of the nano-layered structure and grain boundaries. Grain boundaries act as effective obstacles to dislocation movement, thereby increasing the material’s strength. Another possibility is the heterogeneous deformation-induced strengthening, where the strain gradient formed between different regions during plastic deformation leads to the generation of reverse stress or positive stress, which can enhance the material’s hardening ability. Chen et al.’s research indicates [90] that various tissues present in the sample all contribute to the overall strength to varying degrees. The strength contribution of a single tissue can be estimated using the Hall–Patch method. Among them, the contribution of grain boundary strengthening can be given by $\sigma_{G}=kd^{-\frac{1}{2}}$. In the crystal, there are also geometrically necessary dislocations (GNDs) generated to adapt to the deformation gradient and satisfy the geometric compatibility conditions of crystal plastic deformation. The contribution of high-density dislocations to the overall strength can be given by the Taylor hardening model $\sigma_{D}=M\alpha Gb\rho^{-\frac{1}{2}}$, where ρ is the dislocation density, which can be given by $\rho_{GND}=\frac{2\theta }{\mu b}$. The meanings of the parameters in the above three formulas are as follows: $\sigma_{G}$ is the contribution of grain boundary strength, k is the grain boundary strengthening coefficient, d is the average grain size, $\sigma_{D}$ is the contribution of dislocation strength, M is the Taylor factor, α is the constant of face-centered cubic metals, b is the Burgers vector, G is the shear modulus of SLM-prepared Ni_2.1_, ρ is the dislocation density, θ is the orientation difference angle, and μ is the step length. Through the estimation of the above formulas, it was determined that the contribution of grain boundaries to the yield strength of the SLM-prepared Ni_2.1_ sample is 214 MPa, and the contribution of dislocations to the strength is approximately 287 MPa. However, the stress–strain curve given in Figure 11A indicates that the yield strength of the sample is much higher than the calculated theoretical strength, which is 1329 ± 12 MPa. Therefore, it was determined that the additional strengthening effect comes from the layered structure and precipitated phases existing within the material.

From Figure 11C–F, it can be seen that the surface morphology of the fracture surface of the Ni_2.1_ sample prepared by SLM presents a pattern of fine grooves and ductile pits. This is because during the plastic deformation process, the soft phase FCC is elongated along the strain direction, while the hard phase B2 hardly undergoes deformation. This structure can effectively disperse stress and prevent local stress concentration from leading to premature failure. Moreover, the interface between the layered structure composed of FCC and B2 can effectively prevent dislocations from passing through, thereby increasing the material strength. Additionally, due to the rapid cooling process, the formed lamellar structure is relatively fine, resulting in more grain boundaries, which further enhances the material’s strength. During plastic deformation, the gradient of strain between different phases will further amplify the obstruction effect of grain boundaries on dislocation movement, significantly enhancing the mechanical properties of the material.

**Figure 12:**
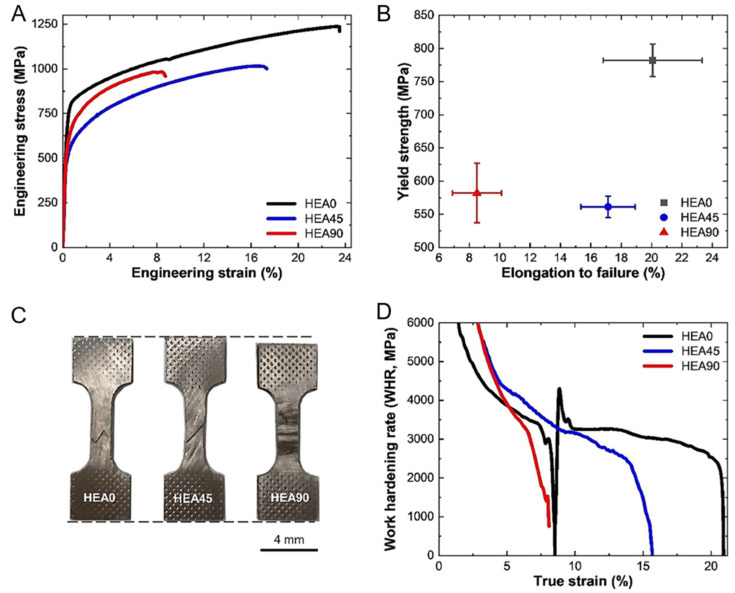
(**A**) Engineering stress–strain curves of samples HEA0, HEA45 and HEA90, (**B**) comparison of yield strength and fracture elongation of samples HEA0, HEA45, and HEA90, (**C**) macroscopic images of the three specimens after tension, (**D**) relationship between WHR and true strain of samples HEA0, HEA45, and HEA90, adapted from reference [92].

Chen, Lang et al. took three parallel samples with different orientations from the same sample, as shown in Figure 5A, namely HEA0, HEA45, and HEA90. They tested their mechanical properties. Figure 12 presents the engineering stress–strain curves, yield strength, macroscopic images after fracture, and work hardening rate of the three samples.

The HEA0 sample exhibited the highest yield strength and tensile ductility due to its loading direction being parallel to the nanosheet structure within the overall structure. Its yield strength was approximately 800 Mpa and its tensile ductility was 23.5%. In contrast, the HEA45 sample had the lowest yield strength, approximately 580 Mpa, and moderate tensile ductility, approximately 17.2%. The HEA90 sample had a moderate yield strength, approximately 625 Mpa, and the lowest tensile ductility, approximately 8.5%.

**Figure 13:**
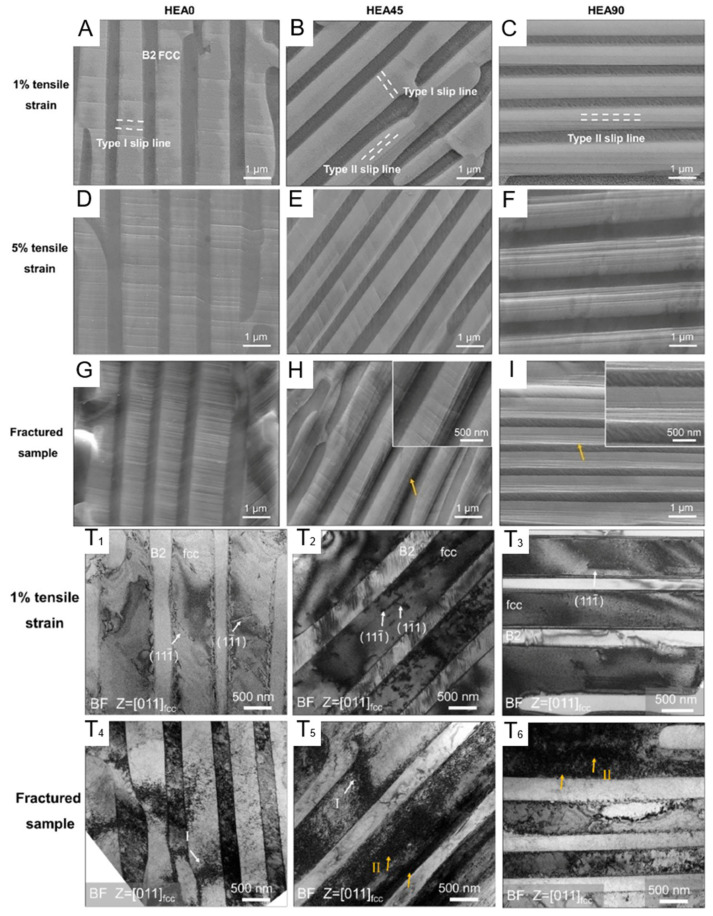
SEM images of different slip line activation situations for the three samples, and TEM images after different strains. (**A**) SEM image of HEA0 sample after 1% strain, (**B**) SEM image of HEA45 sample after 1% strain, and (**C**) SEM image of HEA90 sample after 1% strain. (**D**) SEM image of HEA0 sample after 5% strain, (**E**) SEM image of HEA45 sample after 5% strain, and (**F**) SEM image of HEA90 sample after 5% strain. (**G**) SEM image of HEA0 sample near the fracture point after fracture, (**H**) SEM image of HEA45 sample near the fracture point after fracture, and (**I**) SEM image of HEA90 sample near the fracture point after fracture (the upper right corner of (**H**,**I**) shows the stacking situation of slip lines at the phase boundary). (**T1**–**T3**) are TEM images of HEA0, HEA45, and HEA90 samples after 1% strain, and (**T4**–**T6**) are TEM images of HEA0, HEA45, and HEA90 samples near the fracture surface after fracture, adapted from reference [92].

Figure 13 shows the SEM (Figure 13A–I) and TEM Figure 13(T1–T6) images of different samples after deformation under different strains. In the study, two slip systems were artificially defined [92]. The slip lines perpendicular to the phase interface are classified as Type-I, and those parallel to the phase interface are classified as Type-II. In the HEA0 sample, only Type-I slip lines exist, while in the HEA45 sample, both Type-I and Type-II slip lines coexist, and in the HEA90 sample, only Type-II slip lines exist. As the strain increases, the spacing of the slip lines in the HEA0 sample decreases and becomes more uniform. In the HEA45 sample, the number of Type-I slip lines exceeds that of Type-II slip lines, and they are more uniformly distributed. In the HEA90 sample, the distribution of Type-II slip lines is uneven, and they accumulate at the phase interface after fracture, enhancing the crack initiation tendency. Figure 13(T1,T5) indicates that after fracture of the HEA0 sample, a high-density Type-I dislocation is formed. This adaptive deformation helps to improve the overall strength of the material. In the HEA45 sample, there are Type-I dislocations and high-density Type-II dislocations. In the HEA90 sample, there are high-density Type-II dislocations that are accumulated at the phase interface, restricting further plastic deformation.

In contrast, due to the uniformly distributed high-density dislocations within the HEA0 sample, its overall performance is superior to that of the HEA45 and HEA90 samples. This result further proves that specific microstructures in the material have a significant impact on the overall performance. Combining this with Figure 12C, it shows that the fracture surface of the HEA0 sample is serrated, while the fracture surfaces of the other samples are straight. This indicates that when loading along the lamellar direction, the internal stress distribution of the material is more uniform, reducing local stress concentration. Comparing the yield strengths of the samples from other two angles, it can be determined that the loading direction relative to the nano-layered structure significantly affects the activation of slip systems and the distribution of dislocations. This also indicates that the layered structure has significant anisotropy in enhancing performance. This provides an effective reference for the subsequent design of more superior layered heterogeneous structure materials.

### 4.3. Additive Manufacturing of Multi-Level Heterogeneous Structures

The multi-level heterogeneous structure not only has the individual effects of various strengthening structures, but also the combined effects among multiple structures. Its deformation mechanism is diverse. For example, in samples without aging treatment, due to factors such as dynamic grain refinement, dislocation strengthening, and precipitation strengthening, the alloy exhibits high dynamic shear strength and ductility synergy. Planar dislocation slips cause additional strengthening effects. The precipitated phases distributed along grain boundaries during annealing help form non-homogeneous lamellar structures. Solid solution strengthening occurs during deformation. Mechanical twinning appears during deformation, generating twin-induced plastic strengthening effect (TWIP), and heterogeneous deformation-induced strengthening (HDI). These studies show that the multi-level heterogeneous structure can enable high-entropy alloys to exhibit excellent performance under dynamic loading conditions [101,102,103,104].

**Figure 14:**
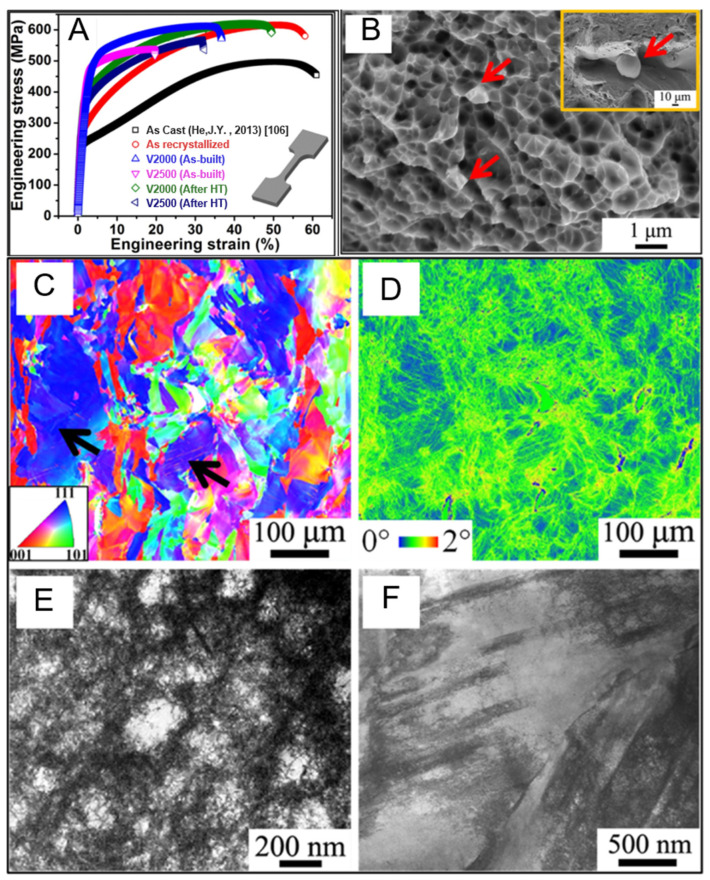
(**A**) Engineering stress–strain curves of the samples under different conditions [106], (**B**) fracture morphology of the original V2000, (**C**) EBSD-IPF diagram, (**D**) KAM diagram, (**E**,**F**) bright-field STEM images of the original state samples that were stretched to fracture, adapted from reference [91].

Figure 14A shows the engineering stress–strain curve of the CoCrFeNiMn high-entropy alloy sample prepared by Zhu et al. through SLM. The V2000 sample prepared exhibits an excellent combination of strength and ductility. Compared to the samples prepared by casting, the yield strength before heat treatment has significantly increased (yield strength $\sigma_{y}$ = 510 ± 10 MPa). After heat treatment, the crystal structure changes, and the yield strength decreases (381 ± 8 MPa), but it is still higher than the yield strength of the cast state (205 ± 5 MPa). However, the ductility increases after heat treatment. Figure 14B shows the fracture surface of the V2000 sample. This morphological characteristic indicates that the fracture may start from the cell wall [105], and the arrow in the figure indicates the presence of nanoparticles within the fracture surface. The component analysis of these nanoparticles shows that they are chromium-rich compounds. These hard particles may become crack sources or stress concentration points due to their uneven distribution or formation of large aggregations during deformation. Figure 14F shows the bright-field STEM image, which reveals the interaction between the slip band and the cellular structure, forming a complex dislocation configuration, effectively hindering the movement of dislocations. These complex dislocation structures effectively enhance the strain hardening ability of the material, enabling the material to maintain stable strain hardening ability under high stress levels.

**Figure 15:**
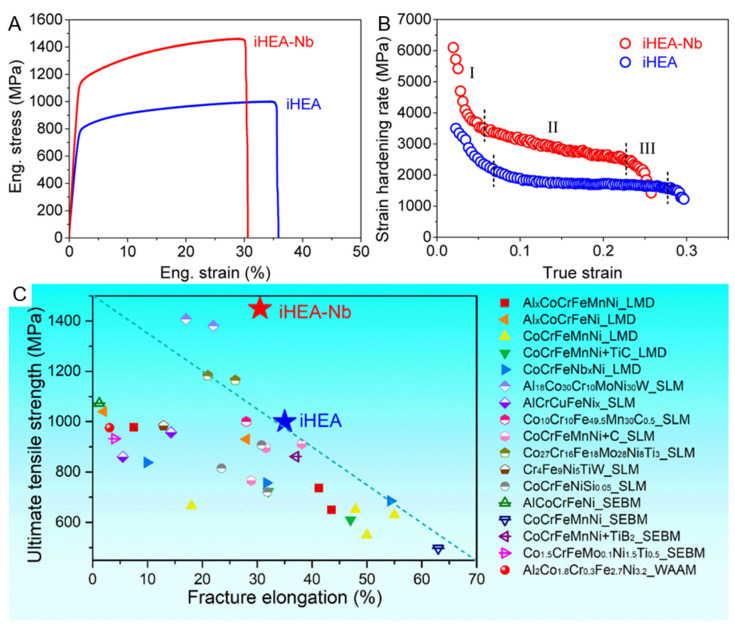
(**A**) Engineering stress–strain curves of iHEA-Nb and iHEA tensile specimens obtained by laser cladding deposition, (**B**) strain hardening rate curves, (**C**) comparison of tensile strength and elongation of HEA prepared by different additive manufacturing techniques, adapted from reference [93].

The metal materials prepared by additive manufacturing usually have a heterogeneous microstructure with a wide range of scales, ranging from a few nanometers to several hundred micrometers. Combined with the added trace elements, they are more likely to form complex microstructures, thereby enhancing the material’s properties. Zhang et al. prepared the iHEA-Nb sample through LMD, and this sample also demonstrated a relatively high level of mechanical performance. Figure 15 shows the engineering stress–strain curve, strain hardening rate curve of the sample, as well as the comparison images with the tensile strength and elongation of different additive manufacturing techniques-prepared HEAs.

From the images, it can be seen that the iHEA-Nb sample exhibits excellent yield strength and tensile strength of 1140 MPa and 1450 MPa, respectively. Compared with the iHEA without Nb addition, these values have increased by 44% and 45%, respectively. Although the improvement in mechanical properties is accompanied by a decrease in the overall elongation rate, the elongation rate still remains at around 30%. By comparing with other components or samples prepared by additive manufacturing methods shown in Figure 15C, iHEA-Nb demonstrates an excellent strength–plasticity synergy effect. The reason for this is that the addition of Nb leads to severe lattice distortion, increases the dislocation density, and the in-situ formed NbC and other carbides can effectively hinder dislocation movement, increasing the strength and toughness of the material. The high-density dislocations and dislocation entanglements formed around precipitates give the material a high strain hardening ability. The different stages of the strain curve correspond to different deformation mechanisms. In stage I, the SHR value (strain hardening rate) decreases due to dynamic recovery causing strain softening. At this time, the deformation mechanism is mainly dislocation slip. In stage II, iHEA-Nb and the iHEA samples show different trends of change. This is because the samples with Nb addition are affected by the solute strengthening effect and precipitation strengthening effect during the deformation process, resulting in more significant dislocation increment and dislocation entanglement phenomena. Therefore, this enhancement effect makes the iHEA-Nb sample show a significantly stronger SHR value at this stage.

**Figure 16:**
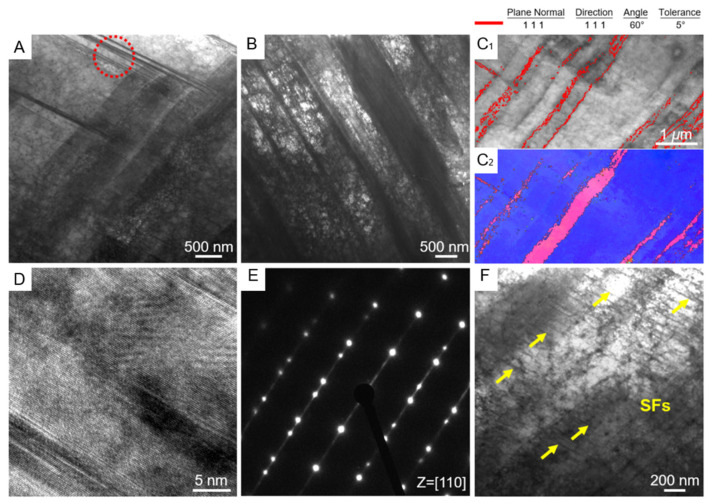
(**A**,**B**) Two thin slices prepared by FIB, their overall bright-field STEM images; (**C**) the image quality (**C_1_**) and IPF diagram (**C_2_**) obtained through transmission EBSD analysis; (**D**) HRTEM image; (**E**) the marked area in (**A**) corresponding to the SAED pattern; (**F**) multiple stacking dislocations found in the FCC matrix phase, adapted from reference [93].

Zhang et al. discovered deformation twinning and stacking faults in the deformed samples, as shown in Figure 16A,B. The samples used for TEM analysis were taken from the region with a strain degree of approximately 30%. The images show that there are a large number of dislocation grids between the stripe structures of the deformed FCC grains. The EBSD analysis in Figure 16C confirmed that these stripe structures are deformation twinning, indicating that the TWIP occurred during the test. When stress is applied, mechanical twinning will form inside the material, and the twin boundaries can hinder the subsequent dislocation movement, causing the dislocation movement path to change, thereby increasing the material strength. In addition, the formation of deformation-induced twinning also enhances the dislocation storage and strain hardening ability of the alloy [105].

**Figure 17:**
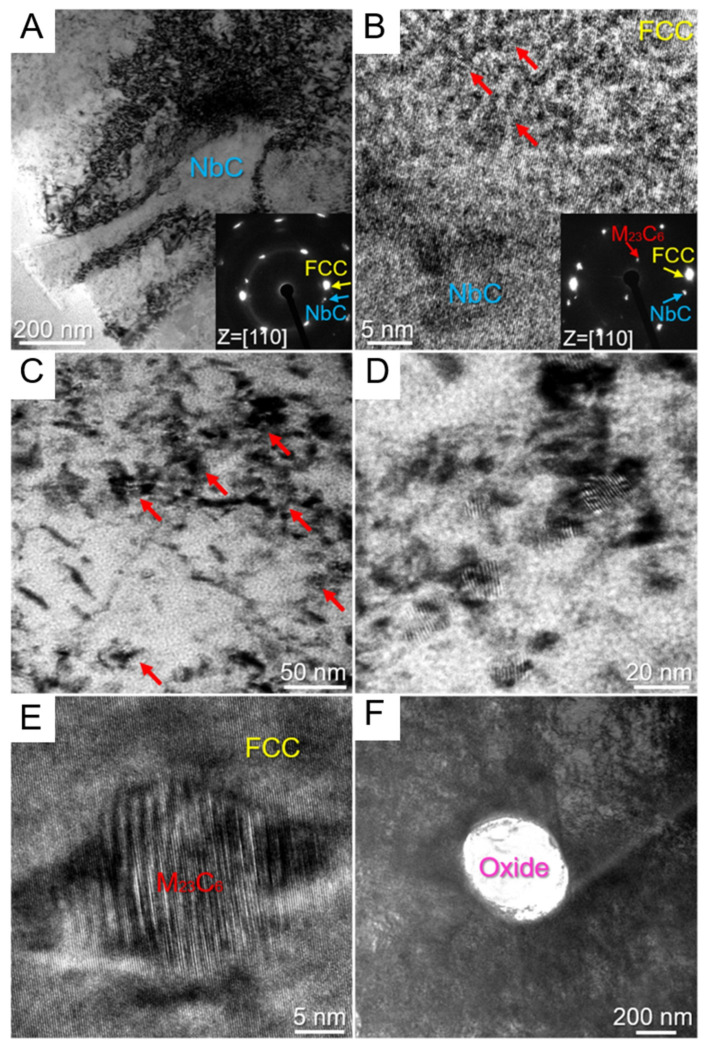
(**A**) TEM image, (**B**) HRTEM image of the NbC interface with the FCC matrix, (**C**) bright-field TEM image of the carbides in the matrix, (**D**) magnified image of the carbides after tensile deformation, (**E**) HRTEM image of the carbides, (**F**) particles of enriched Mn oxide in the deformed area, adapted from reference [93].

Figure 17 shows significant dislocation entanglement around the NbC carbide. Besides the NbC precipitates, a large number of M_23_C_6_ and M_7_C_6_ nanoparticles were also observed (Figure 17B). Although their ability to hinder dislocation movement is not as strong as the NbC precipitates, dislocation aggregation can still be observed around these nanoparticles (Figure 17C). Therefore, they can still exhibit an effective strengthening effect. These hard particles (or second-phase particles) that hinder dislocations are also known as the Orowan precipitation mechanism. The high-density dislocation loops around the nanoparticles form multiple stationary points in the sample, further increasing the difficulty of dislocation movement. However, this effect will also slightly reduce the material’s ductility. Nevertheless, it can significantly enhance the material’s work hardening ability and overall mechanical properties.

## 5. Conclusions and Prospects

Through the analysis of the aforementioned studies, we gain insight into the regulatory effects of microscopic defects in crystals—such as vacancies, dislocations, and secondary phase precipitates—on material properties. It is evident that microstructural features exert a wide range of influences on the intrinsic performance of materials. This is particularly pronounced in complex alloys such as high-entropy alloys (HEAs), where the interplay among various microstructures can lead to highly unpredictable property variations. In Section 2.2, several studies demonstrate the enhancement of mechanical properties in HEAs through the deliberate introduction of defects [5,58,60,61,63], or via transformation-induced plasticity to improve both strength and ductility. The diverse strategies of defect engineering have broadened the research frontiers of HEAs. However, this high degree of design flexibility comes at a considerable cost. As a result, certain studies have begun to explore data-driven machine learning algorithms to predict the microstructural evolution of high-entropy alloys, offering a novel and promising approach for future HEA research [54,57,81,82].

The performance changes brought about by heterogeneous microstructures in AM-HEAs have provided significant inspiration for the future research and application of AM-HEAs. The analysis results of AM-HEAs through various characterization methods (SEM, EBSD, EDS, TEM, XRD, mechanical stretching, nanoindentation, etc.) indicate that by regulating grain size, phase distribution, interface characteristics (low-angle grain boundaries, nano-twinned crystals), as well as introducing nano-precipitates or chemical short-range order, the strength, toughness, and other properties of the material can be significantly improved. The additive manufacturing technologies based on DED and PBF, with their unique local high temperature gradients, high cooling rates, and flexible process parameters, provide new approaches for constructing multi-level, gradient, and localized heterogeneous structures.

At the same time, some new problems have also emerged simultaneously, including the following:

(1) It is necessary to establish the connection between multi-dimensional microstructural defects and mechanical properties. Previous studies have demonstrated that the introduction of microstructural defects can to some extent enhance the mechanical properties of metal or alloy materials. However, the introduction of defects still dominates within the material, meaning that the influencing factors of the strengthening and toughening mechanism are relatively single. It is still difficult to achieve the simultaneous introduction of metastable structures dominated by multi-dimensional defects and the simultaneous attainment of high strength and plasticity, and the correlation of multi-dimensional defects with macroscopic mechanical properties remains unclear. Can we combine the inherent characteristics of high-entropy alloys and select additive manufacturing and other processes to achieve the superposition of multi-dimensional defects, and efficiently prepare high-entropy alloys with excellent comprehensive mechanical properties that combine multi-dimensional microstructural defects?
(2) Interaction and metastable characteristics of microstructural defects. The formation of defect structures is mainly caused by the change in external conditions, resulting in the collapse of the intrinsic state of the material. That is, the atoms exhibit irregular local arrangements. As the external conditions continue to change, the defect intrinsic state eventually shows a superposition. Can the rational spatial distribution and configuration of multi-dimensional microstructural defects be precisely controlled, and can the intrinsic relationship between multi-dimensional microstructural defects and mechanical properties be established? However, how do we achieve the stable superposition of multi-dimensional defects under specific compositions and process conditions, and obtain complex spatial arrangements?
(3) The influence laws of the cooperative deformation mechanism of microstructural defects. At present, there are no reports on the influence laws of the cooperative deformation mechanism of defect microstructures and the deformation interaction mechanism among various defects. In conclusion, related research should be a hot topic in the field of face-centered cubic structure high-entropy alloys in the coming years.
(4) How can the parameters be adjusted to achieve more precise energy input during the manufacturing process, and can a controllable temperature gradient be utilized to achieve the directional design and stable regulation of heterogeneous microstructures, thereby fully exploiting the performance potential of HEAs?
(5) For high-entropy alloys, adjusting their composition is an effective way to modify their properties. However, the composition of high-entropy alloys is highly flexible, and the combinations of various elements are diverse. Designing the composition to directly control the properties of the alloy is quite challenging and costly for the current preparation techniques and control methods. Therefore, the search for effective multi-microstructure co-regulation methods and the exploration of the co-deformation mechanisms of multiple types of microstructures are also urgent issues to be addressed at present.
(6) Although there are reports on the research of additive manufacturing of high-entropy alloys using artificial neural networks at present, how to establish a large-scale model that can predict the microstructure obtained from additive manufacturing of high-entropy alloys with unknown components under multiple computational theories is still a rather difficult problem that needs to be solved at present.

This review not only provides an effective experimental and theoretical foundation for further understanding the precise regulation of the defect structure spatial configuration in high-entropy alloys and the study of the deformation mechanism of various types of microstructural defects in high-entropy alloys, but also has significant theoretical and practical significance for the engineering application of additive manufacturing technology in the field of high-entropy alloys. It will also establish a feedback relationship between additive manufacturing processes, microstructural defects, and mechanical properties. Ultimately, it will achieve efficient and low-cost production of high-entropy alloy materials with excellent performance, effectively promoting the application of high-entropy alloys in key components in fields such as national defense and aerospace.

## Figures and Tables

**Table 1:** Mechanical properties of samples prepared by different methods and with different components.

Method	Composition	Microstructure	YS (Mpa)	UTS (Mpa)	HV	PS (%)
WAAM [86]	AlCoCrFeNi (21%Al)	Bottom BCC + FCC	827.4 ± 15.1	2720.8 ± 29.4	342.3 ± 9.4	42.3 ± 0.7
	AlCoCrFeNi (30%Al)	Middle BCC + FCC	877.1 ± 21.3	2047.9 ± 18.5	370.6 ± 13.7	24.5 ± 1.8
	AlCoCrFeNi (35%Al)	Top BCC	955.5 ± 5.4	1712.0 ± 38.6	369.9 ± 7.3	17.1 ± 0.7
LPBF [89]	AlxCoCrFeNi (x = 0.04–0.75)	BCC + FCC	560–640	700–760	220–550	1.33–24
SLM [90]	AlCoCrFeNi_2.1_	B2 + FCC	1329 ± 12		-	
SLM [91]	CoCrFeNiMn	Multi-level heterogeneous structure	510 ± 10		-	
LDED [92]	AlCoCrFeNi_2.1_-HEA0	FCC + B2	800		-	23.5
	AlCoCrFeNi_2.1_-HEA45		580		-	17.2
	AlCoCrFeNi_2.1_-HEA90		625		-	8.5
LMD [93]	Fe_49.5_Mn_30_Co_10_Cr_10_C_0.5_	Multi-level heterogeneous structure	1140	1450	-	
LDED [87]	AlCoCrFeNi_2.1_	B2 + FCC	773 ± 18	1214 ± 20	-	16.3 ± 0.6
LDED [88]	CoCrFeNiMo	FCC + σ+μ	-	-	658.44	-

**Table 2:** The mechanical properties of Al-HEA were adapted from reference [86].

Region	Hardness (HV)	YS (MPa)	FS (MPa)	PS (%)
Top	369.9 ± 7.3	955.5 ± 5.4	1712.0 ± 38.6	17.1 ± 0.7
Middle	370.6 ± 13.7	877.1 ± 21.3	2047.9 ± 18.5	24.5 ± 1.8
Bottom	342.3 ± 9.4	827.4 ± 15.1	2720.8 ± 29.4	42.3 ± 0.7
